# Reduced Food Availability Amplifies Behavioral Depression and Impairs Locomotor Performance After Immune Challenge in *Xenopus laevis*

**DOI:** 10.1002/jez.70119

**Published:** 2026-08-03

**Authors:** Thaysa G. Oliveira, Laurie Araspin, Laura Camila C. Olarte, Carlos A. Navas, Anthony Herrel

**Affiliations:** ^1^ Department of Ecophysiology and Evolutionary Physiology University of São Paulo USP São Paulo Brazil; ^2^ Département Adaptation du Vivant UMR 7179 C.N.R.S./M.N.H.N. Paris France; ^3^ Naturhistorisches Museum Bern Bern Switzerland; ^4^ Department of Biology, Evolutionary Morphology of Vertebrates Ghent University Ghent Belgium; ^5^ Department of Biology University of Antwerp Wilrijk Belgium

**Keywords:** amphibians, caloric restriction, energy trade‐offs, immune challenge, locomotion, sickness behavior

## Abstract

Mounting an immune response and locomotor activity are energetically costly processes that can generate trade‐offs affecting energy allocation, muscle performance, and activity patterns. The balance between these demands can be modulated by factors such as food availability and ecological conditions, influencing the ability to mount an immune response and maintenance of locomotor activity. In a previous study, we demonstrated that *Xenopus laevis* subjected to an immune challenge maintained voluntary movement, indicating that behavioral depression did not occur in these animals, contrary to what is observed in most species. We hypothesized that food availability influenced this response, since the animals were captive and fed *ad libitum*. Here, we investigated the effects of food restriction on the locomotor activity of *X. laevis* after an immune challenge induced by lipopolysaccharide (LPS). We evaluated locomotor endurance, jump force, and spontaneous voluntary movement in individuals subjected to a progressive, 30‐day, food restriction protocol. These results suggest that food restriction alters the energetic prioritization following immune activation, leading to reduced locomotor output and behavioral activity. Furthermore, the reduction in jump force and locomotor endurance was more pronounced in individuals on restricted diet, indicating a direct impact of energy availability on locomotor capacity. These findings reinforce the idea that resource availability influences the immune and locomotor response in anurans, highlighting how nutritional stress may constrain immune‐locomotor trade‐offs in amphibians, with potential consequences for survival and fitness in resource‐limited habitats.

## Summary

Calorie restriction increases behavioral depression and reduces locomotor capacity after immune challenge in Xenopus laevis, revealing how energy limitation shapes tradeoffs between immune performance and activity.

## Graphical abstract

Food restricition amplifies the reduction in voluntary locomotion after immune challenge in Xenopus laevis, ilustrating the energetic trade‐off between immunity and performance.

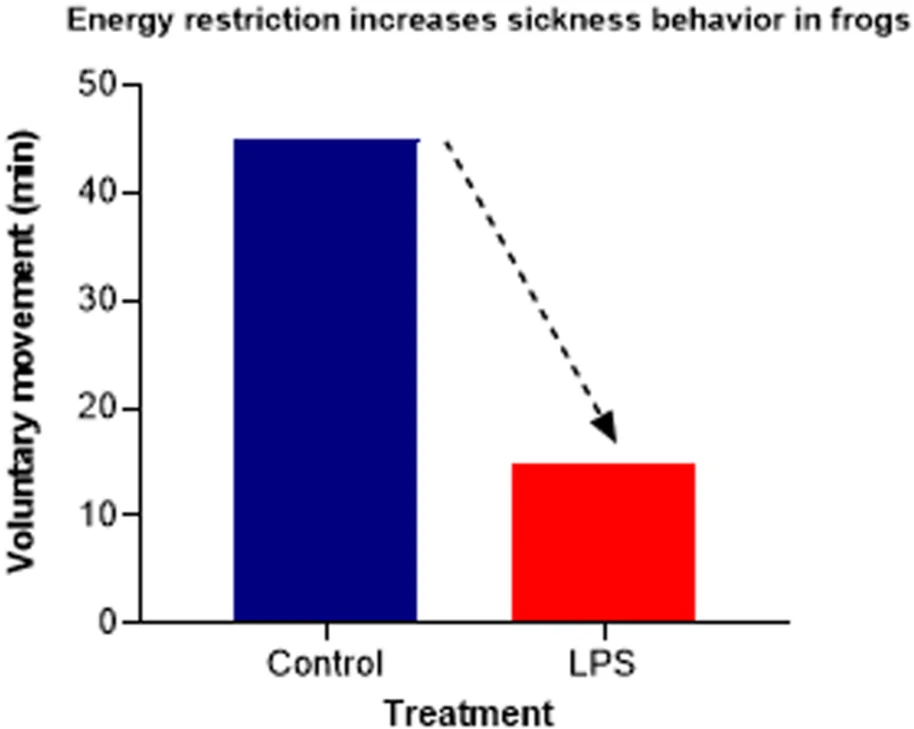

## 1 Introduction

Locomotion is a fundamental aspect of survival for most vertebrates, playing an essential role in activities such as searching for food, escaping from predators, and seeking reproductive partners (Taigen et al. 1982; Taigen and Pough 1985; Dickinson et al. 2000). However, it requires considerable energetic and metabolic resources, which may compete with other physiological demands (Taigen and Pough 1981; Taigen et al. 1982; Pough et al. 1992), including immune responses (Muchlinski 1985; Hart 1988; Sheldon and Verhulst 1996; Husak et al. 2016). This reflects energy allocation decisions, organisms operate under limited energy budgets and must sustain competing physiological processes, such as maintenance, growth, reproduction, and immune function (Sheldon and Verhulst 1996; Zuk and Stoehr 2002; Husak et al. 2016). These competing needs result in compromises often involving locomotion, and where energy investment in one function occurs at the cost of others (Hart 1988; Adelman and Martin 2009; Husak et al. 2016). These trade‐offs become particularly relevant when organisms face stress, for example as part of an immune challenge, or other factors generating limited energy availability. The resulting balance may constrain performance and alter behavior (Hart 1988; Husak et al. 2016), including the locomotion component of it.

This energetic trade‐off can cause reduced locomotion, as part of a broader behavioral depression, characterized by reduced activity and social interactions which is common following a severe immunological challenge. This phenomenon is well documented in several groups of vertebrates, both endothermic (Hart 1988; Dantzer 2006; Adelman and Martin 2009) and ectothermic (Llewellyn et al. 2011; Braga 2013; Oliveira 2022; Oliveira et al. 2024; Oliveira et al. 2025), and is considered adaptive in the sense that it favors recovery by prioritizing the energy to mounting an immune response. However, these trade‐offs may not be universal in either nature or scope. Some species seem able to modulate or limit the impact of immune challenge on behavior, particularly those with high dispersal capacity (Brown and Shine 2014; Gardner et al. 2020; Oliveira et al. 2024).

Despite the broad nature of behavioral depression, locomotion has been a dominant measure because it is ecologically essential, energetically demanding, and experimentally tractable, and is thought to reflect key energetic trade‐offs, where reductions in movement signal the reallocation of metabolic resources (Husak et al. 2016; Llewellyn et al. 2011; Gardner et al. 2020; Oliveira 2022; Oliveira et al. 2024). The physiological demands of immune activation can directly impair locomotor capacity, particularly when energy availability is limited (Sherman & Stephens 1988; Sheldon and Verhulst 1996; Zuk and Stoehr 2002; Husak et al. 2016). During an immune response, metabolic resources are redirected toward the synthesis of inflammatory mediators and other immune effectors, which can reduce physical performance (Dantzer 2004; Dantzer et al. 2006; Ferreira et al. 2021; Bastos et al. 2022). This trade‐off becomes even more pronounced under food restriction (a possible consequence of depressed behavior) or additional environmental stress, cases in which conserving energy becomes critical for survival (Pough et al. 1992; Oliveira et al. 2025). Investigating how different species navigate these physiological demands may shed light on interspecific variation in life‐history strategies, energetic allocation, and ecological resilience.

In a previous study with *Xenopus laevis*, we investigated how immune system activation influences behavior in anurans, using lipopolysaccharide (LPS) treatment as a proxy for infection and assessing behavior through measures of locomotor endurance, jump force, and voluntary movement (Oliveira et al. 2024). Under such treatment, frogs exhibit reduced locomotor endurance and jump force, but preserve voluntary movement, suggesting limited behavioral depression. These results contrast with more typical reductions in voluntary activity, a pattern observed across several vertebrate taxa during immune challenges (Hart 1988; Dantzer 2006; Llewellyn et al. 2011). Thus, we hypothesized that the maintenance of voluntary activity in our previous study may be related to captive conditions in which food was available *ad libitum*, reducing the need for energy conservation during immune activation. To test this, we conducted a new experiment incorporating a food restriction protocol prior to the simulated infection. Our aim was to assess whether energy availability influences the locomotor response of anurans to an immune challenge. We focused on the same performance variables as before—locomotor endurance, jump force, and voluntary movement—to evaluate whether reduced food supply would exacerbate the decline in locomotor activity, particularly spontaneous movement, as a strategy to conserve energy.

## 2 Methodology

### 2.1 Study Animals

Juvenile African clawed frogs (*Xenopus laevis*; Daudin, 1802) used in this study were obtained from a captive‐bred population at the Muséum National d'Histoire Naturelle in Paris, France (MNHN). These individuals were descendants of wild‐caught animals collected from a single lake near the invasion center in the Deux Sèvres department, France. Native to South Africa, *X. laevis* has established invasive populations in several parts of the world due to accidental or intentional laboratory releases (Lillo et al. 2005; Fouquet and Measey 2006). Animals were housed separately by sex in automated water recirculation systems (Xenrack, Aquatic enterprises) in a room maintained at 22°C, considered the ideal temperature for the species (Casterlin and Reynolds 1980; Miller 1982). All individuals were maintained under a 12 h light: 12 h dark cycle and were fed twice a week with cubes of beef heart during the light phase, preserving a protocol that has been maintained for several generations in captivity. Under these conditions, animals become habituated to daytime activity, therefore all behavioral trials were conducted during the light phase.

### 2.2 Animal Maintenance During Testing

Testing was conducted between September and December 2023. For each test, animals were individually transferred to uniform tanks maintained under the same conditions previously described. Each animal was evaluated separately and sequentially over three test days, with 48‐h rest intervals between sessions. Animals were first assessed prior to any injection and, after a 1‐week interval, were tested again following administration of lipopolysaccharide (LPS) in the experimental group or saline solution in the control group.

### 2.3 Body Mass

All animals were weighed before and after each session using an electronic balance (Cgoldenwall Candel CNA‐383H; precision ± 0.1 g).

### 2.4 Food Restriction Protocol

As explained, the standard protocol involved feeding animals twice a week with beef heart (±1.5 g per individual/meal). For the experimental treatment, both control and LPS‐treated groups underwent a progressive reduction in food availability, starting 4 weeks before the start of the tests. During the first 2 weeks, we decreased feeding to once a week, yet maintaining portion sizes. Starting at the third week, we preserved the already reduced feeding frequency and decreased portions to about half original size (±0.75 g per individual/week). During the last week before the tests, animals remain not fed. Therefore, the protocol reduced feeding gradually across 30 days, culminating in a short fasting period.

### 2.5 Administration and Dosage of LPS and Saline

The simulated infection was induced by injecting lipopolysaccharide (LPS) from *Escherichia coli*, serotype O111:B4, purified by phenol extraction, at a dose of 2 mg/kg. The LPS was diluted in sterile saline, and the volume was adjusted for each individual according to body weight. Injections were performed into the dorsal lymphatic sac using a 29‐gauge needle syringe (Bicego and Branco 2002; Llewellyn et al. 2011; Olarte 2017). All injections were administered at 08:00 a.m., followed by testing 1 h later. Control animals received phosphate‐buffered saline (PBS, Sigma‐Aldrich, pH 7.4) in a volume equivalent to that of the LPS group, following the same injection protocol.

### 2.6 Experimental Groups

After the food restriction protocol, individuals were randomly assigned to either the LPS or control (saline) treatment groups. Eighteen female *X. laevis* were injected with LPS, following the same protocol used in the previous experiment (Oliveira et al. 2024; 2025). An additional 18 females received saline solution as a control. For each locomotor variable (endurance, jump force, and voluntary movement), independent groups of six individuals per treatment were used. Thus, each variable was analyzed separately with a total sample size of *N* = 12 (6 control and 6 LPS). Accordingly, no individuals were shared across measurements. Baseline and post‐injection measurements were obtained from the same individuals for each variable. Due to the limited availability of animals, each locomotor variable was assessed in a separate set of individuals, resulting in 36 animals used across the study. To avoid repeated LPS administration in the same subjects, separate animals were used for each test. Moreover, since LPS has a limited duration of physiological effect, all tests were conducted within its known window of maximum response and at standardized times post‐injection. All animals were euthanized at the end of the experiment, following ethical guidelines.

### 2.7 Experimental Arenas

#### 2.7.1 Locomotor Endurance

This performance trait was tested using a circular track measuring 3 m in circumference. The track was filled with 10 cm of water. For testing, animals were placed into the track and encouraged to swim until exhaustion with soft touches on the back. We recorded the total time spent moving (min), using a stopwatch, and the total distance traveled (m) (see Herrel et al. 2012; Herrel & Bonneaud 2012; Herrel et al. 2014).

#### 2.7.2 Jump Force

This variable was tested on a 20 × 10 cm piezoelectric force platform connected to a Kistler charge amplifier (see Herrel et al. 2014 for a detailed description of the set‐up). Individuals were placed on the platform, one at a time, and left to rest for a few seconds. Next, they were encouraged to jump by gentle touching on the body. Forces were recorded at 500 Hz during 60 s recording sessions and frogs were induced to jump multiple times per session. We used the Kistler Bioware software to extract X‐, Y‐ and Z‐forces for each jump, corrected the signals for drift when present, and calculated the final resultant force in newtons unit (N). We ran analyses based on the three best jumps per session (Herrel et al. 2014).

Voluntary movement: We used an open‐field test to quantify voluntary movement. Animals were placed in a large tank (80 × 50 × 40 cm) filled with 20 cm of water and a shelter (Videlier et al. 2015). At the beginning of the test, the animals were placed under the shelter and left in the tank, being filmed in dorsal view for 12 h (8:00 am – 8:00 pm) with a Sony Handycam (HDR‐CX740). The 12‐h recording period was chosen to encompass the entire light phase, allowing for the quantification of spontaneous activity patterns throughout the entire activity period and reducing the potential bias associated with short‐term observations. The videos were analysed manually and the total time spent moving (min), the total time in the shelter (min), and the number of breaths (number of times the animal went to the surface to breathe) (count) were recorded using a stopwatch.

### 2.8 Statistical Analysis

Data were initially tested for normality using the Shapiro‐Wilk test. Variables that deviated from normality were log_10_‐transformed, and normality was reassessed to confirm suitability for parametric tests. To evaluate the potential influence of body mass on the response variables, ANCOVA was used with body mass as a covariate. Since body mass did not significantly affect any of the analyzed variables (all *p* > 0.005; see Table S1), it was excluded from further analyses and results presentation.

We performed a two‐way mixed‐design ANOVA, with treatment (LPS/saline) as a between‐subject factor and test day as a within‐subject factor; and included the interaction between treatment and test day. Next, we performed post‐hoc tests with Bonferroni correction to compare treatments and test days. These included (i) comparisons among test days within each treatment group (e.g., Control Day 1 vs. Day 2 and Day 3), and (ii) comparisons between treatments within each test day (e.g., Control vs. LPS on Day 1).

All analyses were performed in R (RStudio version 4.4.2), and all R packages used, along with the analysis script, are provided in Supporting Material 2.

## 3 Results

### 3.1 Locomotor Endurance

**Figure 1:**
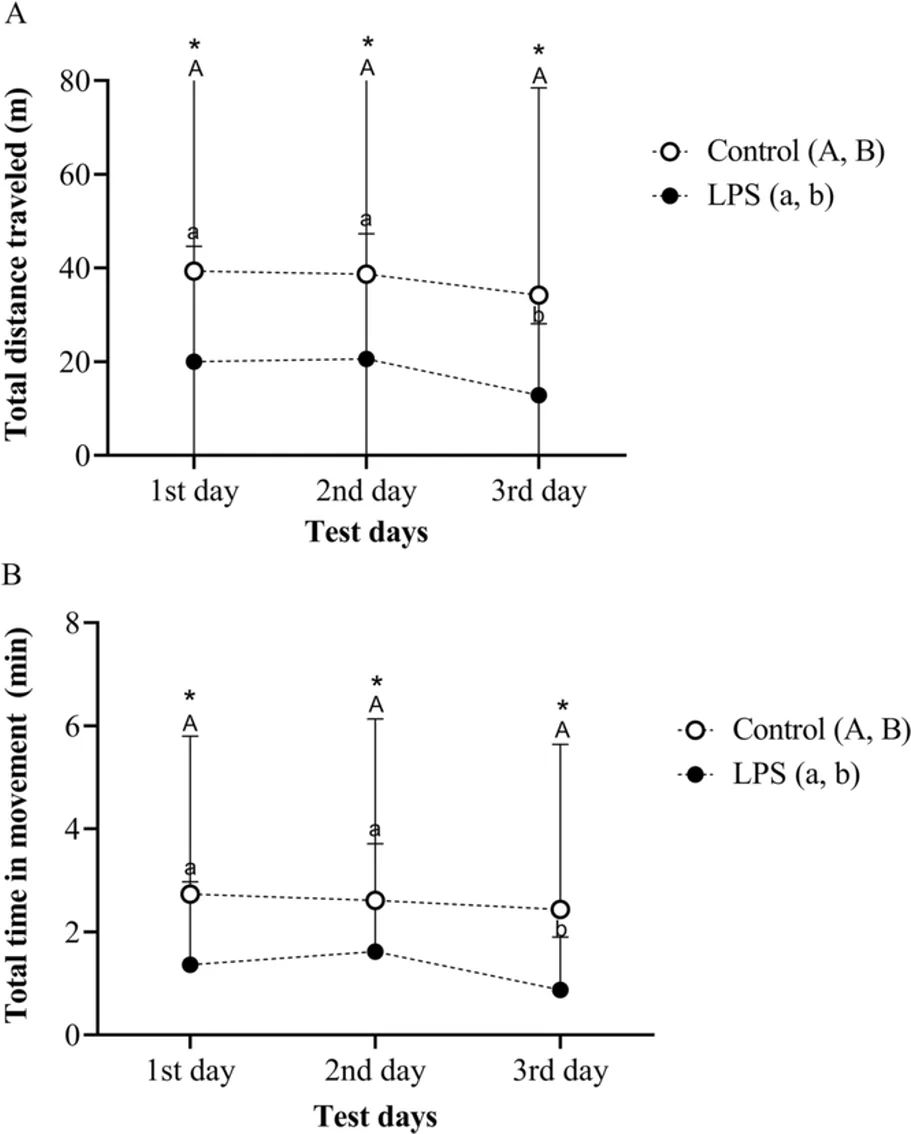
Comparison of locomotor endurance in *Xenopus laevis* injected with saline (control group) or lipopolysaccharide (LPS; treatment group) across three test days. (A) Total time spent moving until exhaustion (min). (B) Total distance traveled until exhaustion (m). Values are presented as mean ± SEM. White circles represent the control group and black circles represent the LPS group. Asterisks indicate significant differences between treatments within the same test day. Different lowercase letters indicate significant differences among test days within the same treatment group (two‐way mixed ANOVA followed by Bonferroni post‐hoc test, *p* < 0.05).

The total distance traveled was reduced by 52.7% in LPS‐injected individuals compared to control animals (*N* = 12, F_1,__11_ = 10.0, *p* < 0.001; Table 1; Figure 1A). Additionally, a significant interaction between treatment and test day was observed after LPS administration (*N* = 12, F_1_,_11_ = 5.34, *p* = 0.018; Table 1). Post‐hoc analysis revealed that, within the LPS group, the distance traveled on the third test day was significantly lower than on both the first and second days, with reductions of 36.8% and 40.2%, respectively (*p* = 0.006 and *p* = 0.001; see Table S2).

**Table 1:** Repeated measures ANOVA test comparing locomotion behavior between Xenopus laevis injected with LPS (treatment group) and saline (control group), on the three test days for each variable analyzed.

Repeated measures ANOVA – Treatment ∗ Locomotor Behavior					
Variable	Comparisons	Mean square	d.f	F	*p*
Locomotor endurance					
Total distance traveled (m)	Treatment	12.58	1.11	18.6	< 0.001
	Test days	46.01	2.10	1.35	0.289
	Treatment ∗ Test days	10.85	2.10	5.34	0.018
Total time spent in movement (min)	Treatment	55.13	1.11	60.2	< 0.001
	Test days	0.962	2.10	2.80	0.093
	Treatment ∗ Test days	2.741	2.10	4.54	0.029
Jump force (N)	Treatment	1.456	1.11	5.79	0.029
	Test days	0.398	2.10	1.58	0.238
	Treatment ∗ Test days	2.239	2.10	14.6	< 0.001
Voluntary movement					
Total voluntary movement time (min)	Treatment	8.281	1.11	35.9	< 0.001
	Test days	6.060	2.10	2.63	0.105
	Treatment ∗ Test days	30.10	2.10	0.10	0.902
Total time inside the shelter (min)	Treatment	1.31e + 6	1.11	67.3	< 0.001
	Test days	9.371	2.10	0.48	0.627
	Treatment ∗ Test days	1.350	2.10	0.66	0.527
Number of breaths (count)	Treatment	2.160	1.11	19.5	< 0.001
	Test days	6.383	2.10	5.23	0.019
	Treatment ∗ Test days	1.343	2.10	12.1	< 0.001

*Note:* Significant differences were accepted at *p* < 0.05; d.f = degrees of freedom.

The time spent moving was also reduced by 51% in the LPS‐treated group compared to controls (*N* = 12, F_1,__11_ = 60.2, *p* < 0.001; Table 1; Figure 1B). A significant interaction between treatment and test day was again observed in the LPS group (*N* = 12, F_1,__11_ = 5.34, *p* = 0.018; Table 1). Bonferroni‐corrected post‐hoc tests indicated that movement time on the third test day was significantly lower than on both the first and second days, with reductions of 36% and 48.3%, respectively (*p* = 0.049 and *p* < 0.001; see Table S2).

### 3.2 Jump Force

**Figure 2:**
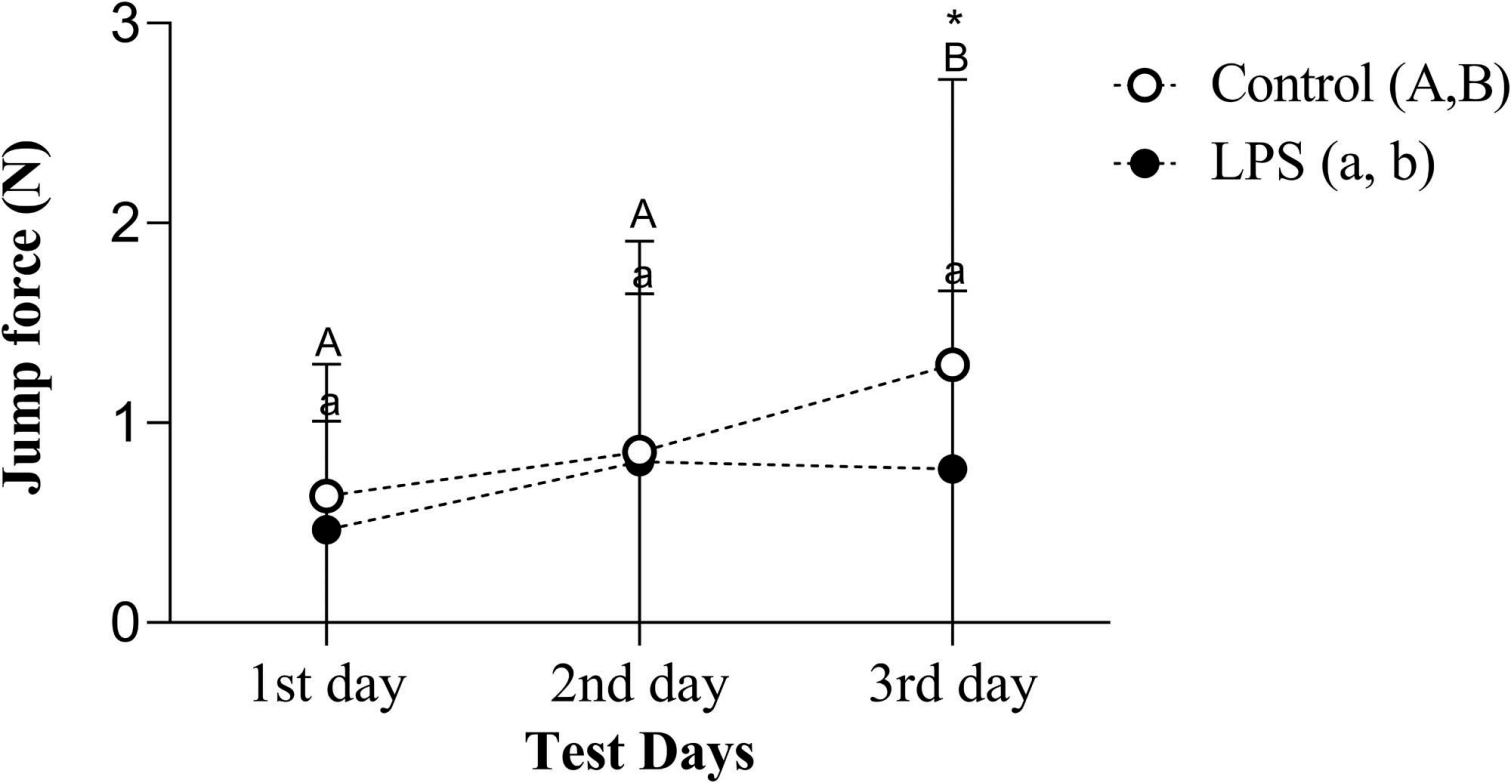
Comparison of jump force (N) in *Xenopus laevis* injected with saline (control group) or lipopolysaccharide (LPS; treatment group) across three test days. Values are presented as mean ± SEM. White circles represent the control group and black circles represent the LPS group. Asterisks indicate significant differences between treatments within the same test day. Different lowercase letters indicate significant differences among test days within the same treatment group (two‐way mixed ANOVA followed by Bonferroni post‐hoc test, *p* < 0.05).

Overall jump force was reduced by 25% in LPS‐injected animals compared to controls (*N* = 12, F_1_,_11_ = 5.79, *p* = 0.029; Table 1; Figure 2). The post‐hoc test demonstrated that the differences between the control and experimental LPS groups occurred only between the third day of testing of the control group in relation to the first (reduction 62.2%), second (36.8% reduction) and third day (36.4% reduction) after LPS (*p* = 0.001, *p* = 0.008, *p* = 0.011; see table S3). Furthermore, there were significant differences in the interaction between treatment and test days (*N* = 12, F_1,11_ = 14.6, *p* < 0.001; Table 1), observed in the control group between the first day of testing in relation to the third day (*p* = 0.021; see Table S3), with a 40% increase in jumping force on the third day of testing.

### 3.3 Voluntary Movement

**Figure 3:**
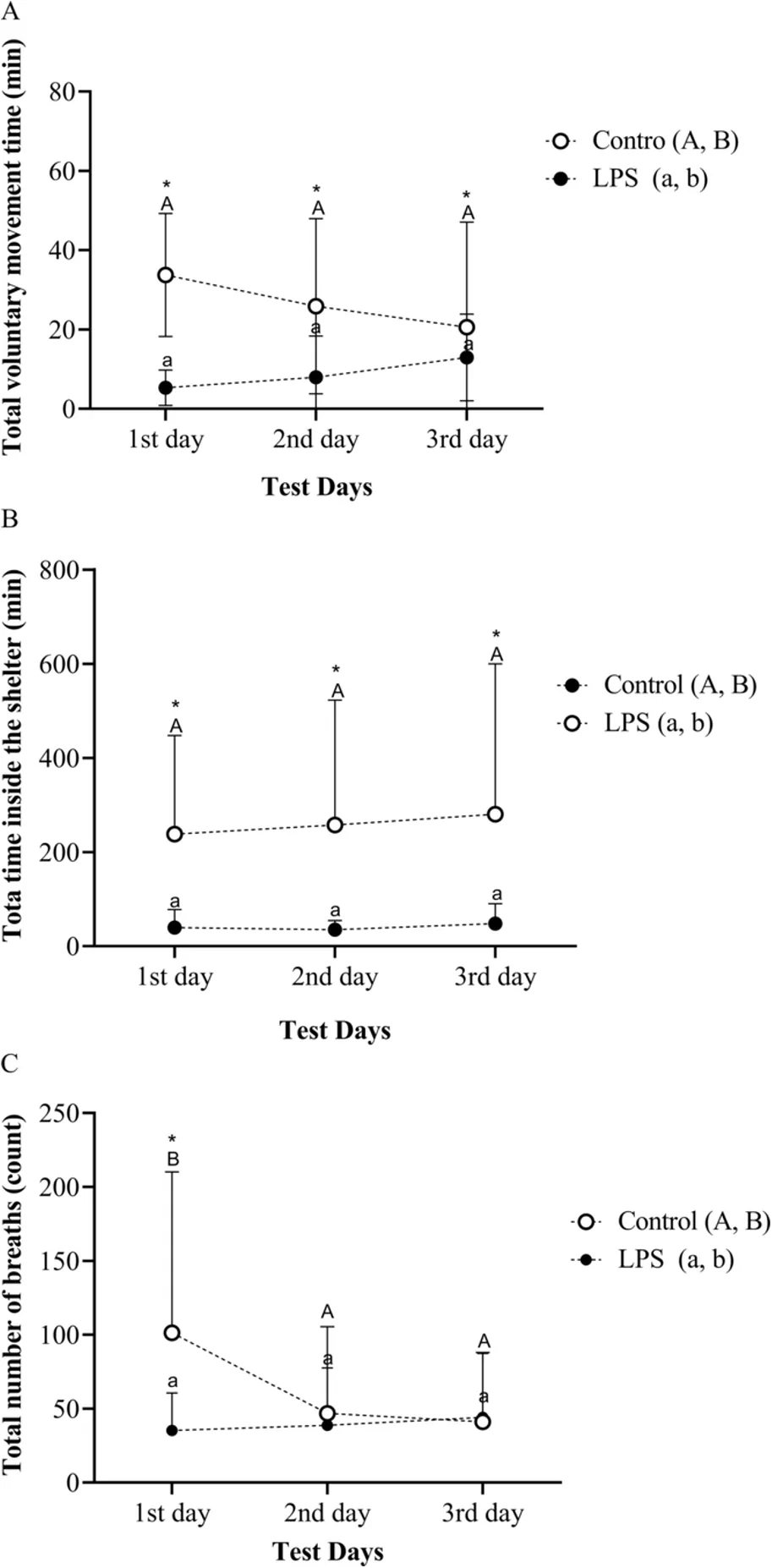
Comparison of voluntary movement in *Xenopus laevis* injected with saline (control group) or lipopolysaccharide (LPS; treatment group) across three test days. (A) Total voluntary movement time (min). (B) Total time spent inside the shelter (min). (C) Total number of breaths (count). Values are presented as mean ± SEM. White circles represent the control group and black circles represent the LPS group. Asterisks indicate significant differences between treatments within the same test day. Different lowercase letters indicate significant differences among test days within the same treatment group (two‐way mixed ANOVA followed by Bonferroni post‐hoc test, *p* < 0.05).

The total time of voluntary movement was 67.2% lower in the LPS experimental group compared to the control group (*N* = 12, F_1.11_ = 35.9, *p* < 0.001; Table 1; Figure 3A), and there were no differences in the interaction between treatment and test days (*N* = 12, F_1,11_ = 0.10, *p* = 0.902; Table 1), confirmed by post‐hoc test (Table S4).

Total time in shelter was 85.3% greater in the LPS experimental group than in the control group (*N* = 12, F_1.11_ = 67.3, *p* < 0.001; Table 1; Figure 3B), without differences between treatment and test days (*N* = 12, F_1,11_ = 0.66, *p* = 0.527; Table 1), also confirmed by post‐hoc test (Table S4).

Contrary to what we observed in the first study, the number of breaths was 40% higher in the control group compared to the LPS experimental group (*N* = 12, F_1.11_ = 19.5, *p* < 0.001; Table 1; Figure 3C). However, these differences were observed only between the first test day in the control group and each of the three test days in the LPS group, with values in the control group being higher by 70.1%, 62.8%, and 58.1%, respectively, compared to the first, second, and third days of testing in the LPS group. Furthermore, there were significant differences in the interaction between treatment and test days (*N* = 12, F_1,11_ = 12.1, *p* < 0.001; Table 1), observed in the control group between the first day of testing in relation to the second and third day (*p* = 0.004, *p* = 0.001; see Table S4), with a reduction in the number of breaths of 50.4% on the second day and 58.3% on the third day.

## 4 Discussion

Our results support the idea of a performance cost associated with immune activation in *X. laevis*, as locomotor performance and voluntary behavior were reduced only under energy‐limited conditions following immune challenge. This contrasts with a similar study under *ad libitum* feeding conditions (Oliveira et al. 2024). Where animals maintained voluntary movement after LPS treatment. These findings indicate that energy is redirected to the immune system, supporting the view that behavioral depression functions as a resource conservation strategy in amphibians (Llewellyn et al. 2011; Braga 2013; Olarte 2017; Oliveira 2022; Oliveira et al. 2024). Consistently, invasive populations already established in the wild, where food is not available ad libitum, also show behavioral depression as the predominant response to infection (Brown and Shine 2014; Goetz et al. 2017; Gardner et al. 2020). Furthermore, unlike what was observed in our previous study, in which no significant differences were observed between test days, LPS treatment in this study had a more pronounced effect on the last test day. This result suggests that, rather than recovery over time, the effects of the immune challenge were progressively intensifying.

Although food restriction is a known strategy to reduce micronutrients such as iron—essential for pathogen growth (Weinberg 2009)—in this case the severity of restriction appears to have outweighed its potential benefits. Although we did not quantify internal energy reserves such as fat body or organ mass, which would provide direct evidence of energy depletion, the observed reductions in locomotor performance are consistent with increased energetic demands following immune activation. The resulting scarcity of energetic resources likely compromised the assembly of an effective immune response, facilitating the progression of the inflammatory condition, as observed in other studies on diet restriction and immune function (Jolly 2004; Venesky et al. 2012; Assis et al. 2023). Jump force was also reduced, which indicating a significant impact on the muscular capacity of individuals subjected to the immunological challenge, in addition to a possible process of energy catabolism in muscle proteins due to food restriction (Neuman‐Lee et al. 2015; Brenes‐Soto et al. 2018). However, this difference was not observed on the first two test days between the control and LPS groups, but only on the third day. Animals in the control group exhibited greater jump force on the third day compared to the first 2 days, suggesting an adaptation effect to the experimental protocol. This indicates that, during the first 2 days, both the control and LPS groups were adjusting to the test, with the control group reaching its maximum performance by the third day. In contrast, animals injected with LPS did not show this improvement, due to the impact of the immune response, which maintained their jump force at a constant level throughout the 3 days. Therefore, the absence of differences between groups in the initial days does not indicate that LPS or the immune response were ineffective; instead, it reflects the possible influence of other factors, such as training and adaptation to the protocol.

Unlike the first study, the control group exhibited a higher number of breaths compared to the LPS‐treated group, but this difference was limited to the first day of testing, progressively decreasing over time. Although immune activation is often associated with increased ventilation, the reduced respiratory frequency observed in LPS‐treated individuals in our study suggests a different physiological response. Moreover, aquatic frogs like *X. laevis* can use skin‐based gas exchanges to ensure oxygen supply when movement is reduced. This pattern may reflect greater voluntary activity and less time spent hiding by the control subjects, given that respiratory rate is closely linked to metabolic demand and increases with aerobic activity (Taigen et al. 1982). In contrast, immune activation in the LPS group may have reduced locomotor activity and overall energy expenditure—potentially through metabolic suppression or energy catabolism—resulting in lower respiratory rates (Larson & Dunn 2001; Brenes‐Soto et al. 2018). These physiological and behavioral changes over the 3‐day testing period may also be influenced by increased levels of stress hormones, such as corticosterone (Bastos 2017; Bastos et al. 2022; Assis et al. 2022). Elevated corticosterone levels after LPS injection have been documented in other models, such as mice, where they modulate exploratory behavior (Carregosa et al. 2024). Corticosterone production is typically intensified by stressors such as prolonged food restriction (Ferreira et al. 2021), and its elevation can increase susceptibility to infection, impair physiological responses, and compromise overall performance (Ferreira et al. 2021; Assis et al. 2023).

It is important to note that the individuals used in this study were immature and, therefore, have a less developed immune system compared to adults (Crespi and Unkefer 2014; Bender et al. 2018; Ruthsatz et al. 2019). This makes them more susceptible to immunological challenges due to the lower efficiency of the immune system (Mikó et al. 2017; Humphries et al. 2022). Furthermore, juveniles have greater energy demands associated with growth (Ferdinand et al. 2020), and the need to balance this expenditure with the immune response (Wright et al. 2023) also explains the observed effects. Moreover, although repeated exposure to the experimental protocol could potentially influence behavioral responses, this explanation is unlikely to account for the observed pattern. Control individuals, which experienced the same procedures, did not exhibit progressive changes across test days. In contrast, LPS‐treated animals showed clear temporal effects, supporting the interpretation that these responses are primarily driven by immune activation rather than protocol‐related adaptation.

One limitation of the present study is the absence of *ad libitum* fed groups in the same experimental design, which would allow for a complete factorial design and a test of the interaction between food availability and immunological challenge. However, previous work using the same species and comparable experimental protocols has shown that *ad libitum* fed individuals do not exhibit behavioral depression as measured by voluntary movement after LPS injection (Oliveira et al. 2024). Thus, the results presented here suggest that reduced food availability plays an important role in modulating the behavioral response to immune activation.

Taken together, our results demonstrate a clear interplay between energy availability, immune activation, and locomotor performance in *X. laevis*. The observed reductions in locomotor endurance, jump force, voluntary movement, and respiratory rate in LPS‐treated, food‐restricted individuals highlight how energetic constraints impose trade‐offs that prioritize immune function at the expense of other physiological and behavioral processes. This energy allocation strategy, while potentially beneficial for survival during infection, results in decreased physical capacity and altered behavior, reflecting a shift in resource investment. Understanding these integrated responses is crucial to understanding how amphibians and other vertebrates balance conflicting physiological demands under environmental stressors, with important implications for their ecology, fitness, and ability to cope with changing environments.

## 5 Conclusion

The results of this study demonstrate that energy availability is a critical factor for the maintenance of locomotor activity following an immune challenge in *X. laevis*. While individuals fed *ad libitum* maintained their movement, those subjected to food restriction exhibited behavioral depression, indicating a mechanism of energy conservation and allocation of resources toward the immune response. These findings reinforce the idea that resource availability influences behavioral patterns and physiology in anurans and provide important insights into the trade‐offs between immunity and locomotion in ecological contexts. Furthermore, the results may have significant implications for the ecology and physiology of invasive species, helping to elucidate how environmental factors modulate their behavioral and physiological responses.

## Author Contributions

Thaysa G. Oliveira conducted the principal experiments. Laurie Araspin helps with experiments, Thaysa G. Oliveira analysed the data. Thaysa G. Oliveira, Anthony Herrel and Carlos A. Navas conceived the study. Thaysa G. Oliveira, L.C, Anthony Herrel and Carlos A. Navas contributed to the writing of the manuscript.

## Ethics Statement

All experiments were performed in accordance with local regulations and approved by the local ethics committee (Comité Cuvier) and Muséum national d'Histoire naturelle‐Paris/FR.

## Conflicts of Interest

The authors declare no conflicts of interest.

## Supporting information


Supporting File

## Data Availability

The files containing the data are stored in the Data Repository of the University of São Paulo USP and can be accessed on the website: https://repositorio.usp.br, searching by the title of the article, name of the authors or keywords. The data are available for access to the general public.
