# Supplementary material for: Reduced Food Availability Amplifies Behavioral Depression and Impairs Locomotor Performance After Immune Challenge in Xenopus laevis
Source: J Exp Zool A Ecol Integr Physiol. 2026 Aug 3;345(8):894–902. doi: 10.1002/jez.70119 (PMC13547877; doi:10.1002/jez.70119)
Supplement: Supplementary file 1 [file JEZ-345-894-s001.docx]

**Supplementary Material**

**Table S1.** One-way ANCOVAs test of the influence of body mass in each variable tested of the locomotor behaviour of *Xenopus laevis* injected with LPS (treatment group) and saline (control group). Each variable was testes separately.

| One-way ANCOVA – Body mass ✻ Locomotor behavior | | | | | | | |
| --- | --- | --- | --- | --- | --- | --- | --- |
| Variable | **Comparisons** | | | **Mean Square** | **d.f** | ***F*** | ***P*** |
| Locomotor endurance |  |  |  |  |  |  |  |
| Total distance traveled (m) | Control | - | LPS | 24.05 | 1.11 | 0.46 | 0.515 |
| Total time spent in movement (min) | Control | - | LPS | 0.005 | 1.11 | 2.12 | 0.179 |
| Jump force (N) | Control | - | LPS | 0.229 | 1.11 | 0.66 | 0.438 |
| Voluntary movement |  |  |  |  |  |  |  |
| Total time in voluntary movement (min) | Control | - | LPS | 44.80 | 1.11 | 1.35 | 0.276 |
| Total time inside the shelter (min) | Control | - | LPS | 10.19 | 1.11 | 0.07 | 0.786 |
| Number of breaths (count) | Control | - | LPS | 17.38 | 1.11 | 0.90 | 0.366 |

Note: d.f degrees of freedom.

**Table S2.** Bonferroni post-hoc test to detect significant differences in locomotor resistance between *Xenopus laevis* treated with LPS (treatment group) and saline solution (control group) on the three test days for each variable analyzed.

| Post Hoc Comparisons - Treatment ✻ Test Days | | | | | | | | | | | |
| --- | --- | --- | --- | --- | --- | --- | --- | --- | --- | --- | --- |
|  | **Comparisons** | | | |  |  |  |  | | |  |
| Variables | **Treatment** | **Days** | **Treatment** | **Days** | **Mean Difference** | **SE** | **df** | | **t** | | ***p* bomferroni** |
|  |  |  |  |  |  |  |  | |  | |  |
| Total distance traveled (m) | Control | 1st day | Control | 2nd day | -2.37 | 6.00 | 15.0 | | -0.394 | 1.000 | |
|  |  |  | Control | 3rd day | 6.92 | 6.00 | 15.0 | | 1.152 | 1.000 | |
|  |  |  | LPS | 1st day | 35.00 | 3.37 | 15.0 | | 10.38 | < .001 | |
|  |  |  | LPS | 2nd day | 32.92 | 4.75 | 15.0 | | 6.924 | < .001 | |
|  |  |  | LPS | 3rd day | 48.83 | 4.75 | 15.0 | | 10.27 | < .001 | |
|  |  | 2nd day | Control | 3rd day | 9.28 | 6.00 | 15.0 | | 1.546 | 1.000 | |
|  |  |  | LPS | 1st day | 37.37 | 4.75 | 15.0 | | 7.860 | < .001 | |
|  |  |  | LPS | 2nd day | 35.28 | 3.37 | 15.0 | | 10.47 | < .001 | |
|  |  |  | LPS | 3rd day | 51.20 | 4.75 | 15.0 | | 10.77 | < .001 | |
|  |  | 3rd day | LPS | 1st day | 28.08 | 4.75 | 15.0 | | 5.907 | < .001 | |
|  |  |  | LPS | 2nd day | 26.00 | 4.75 | 15.0 | | 5.469 | < .001 | |
|  |  |  | LPS | 3rd day | 41.92 | 3.37 | 15.0 | | 12.44 | < .001 | |
|  | LPS | 1st day | LPS | 2nd day | -2.08 | 3.03 | 15.0 | | -0.688 | 1.000 | |
|  |  |  | LPS | 3rd day | 13.83 | 3.03 | 15.0 | | 4.569 | 0.006 | |
|  |  | 2nd day | LPS | 3rd day | 15.92 | 3.03 | 15.0 | | 5.257 | 0.001 | |
| Total time spent in movement (min) | Control | 1st day | Control | 2nd day | -0.252 | 0.50 | 15.0 | | -0.502 | 1.000 | |
|  |  |  | Control | 3rd day | 0.147 | 0.50 | 15.0 | | 0.292 | 1.000 | |
|  |  |  | LPS | 1st day | 2.355 | 0.33 | 15.0 | | 6.954 | < .001 | |
|  |  |  | LPS | 2nd day | 1.727 | 0.39 | 15.0 | | 4.345 | 0.009 | |
|  |  |  | LPS | 3rd day | 3.238 | 0.39 | 15.0 | | 8.149 | < .001 | |
|  |  | 2nd day | Control | 3rd day | 0.398 | 0.50 | 15.0 | | 0.794 | 1.000 | |
|  |  |  | LPS | 1st day | 2.607 | 0.39 | 15.0 | | 6.560 | < .001 | |
|  |  |  | LPS | 2nd day | 1.978 | 0.33 | 15.0 | | 5.842 | < .001 | |
|  |  |  | LPS | 3rd day | 3.490 | 0.39 | 15.0 | | 8.783 | < .001 | |
|  |  | 3rd day | LPS | 1st day | 2.208 | 0.39 | 15.0 | | 5.557 | < .001 | |
|  |  |  | LPS | 2nd day | 1.580 | 0.39 | 15.0 | | 3.976 | 0.018 | |
|  |  |  | LPS | 3rd day | 3.092 | 0.33 | 15.0 | | 9.130 | < .001 | |
|  | LPS | 1st day | LPS | 2nd day | -0.628 | 0.25 | 15.0 | | -2.482 | 0.381 | |
|  |  |  | LPS | 3rd day | 0.883 | 0.25 | 15.0 | | 3.490 | 0.049 | |
|  |  | 2nd day | LPS | 3rd day | 1.512 | 0.25 | 15.0 | | 5.972 | < .001 | |

Note: df= degrees of freedom (using number of pairs of comparisons that were made).

**Table S3.** Bonferroni post-hoc test to detect significant differences in the variable analyzed jump force between *Xenopus laevis* treated with LPS (treatment group) and saline solution (control group) on the three test days.

| Post Hoc Comparisons - Treatment ✻ Test Days | | | | | | | | | | | | | |
| --- | --- | --- | --- | --- | --- | --- | --- | --- | --- | --- | --- | --- | --- |
|  | **Comparisons** | | | | | |  |  |  |  | | |  |
| Variables | **Treatment** | **Days** | **Treatment** | **Days** | | **Mean Difference** | | **SE** | **df** | | **t** | | ***p* bomferroni** |
|  |  |  |  | |  | |  |  |  | |  | |  |
| Jump force (N) | Control | 1st day | Control | | 2nd day | | -0.4400 | 0.292 | 15.0 | | -1.508 | 1.000 | |
|  |  |  | Control | | 3rd day | | -1.1400 | 0.292 | 15.0 | | -3.909 | 0.021 | |
|  |  |  | LPS | | 1st day | | 0.2567 | 0.290 | 15.0 | | 0.886 | 1.000 | |
|  |  |  | LPS | | 2nd day | | -0.3067 | 0.260 | 15.0 | | -1.180 | 1.000 | |
|  |  |  | LPS | | 3rd day | | -0.3233 | 0.260 | 15.0 | | -1.244 | 1.000 | |
|  |  | 2nd day | Control | | 3rd day | | -0.7000 | 0.292 | 15.0 | | -2.400 | 0.447 | |
|  |  |  | LPS | | 1st day | | 0.6967 | 0.260 | 15.0 | | 2.680 | 0.257 | |
|  |  |  | LPS | | 2nd day | | 0.1333 | 0.290 | 15.0 | | 0.460 | 1.000 | |
|  |  |  | LPS | | 3rd day | | 0.1167 | 0.260 | 15.0 | | 0.448 | 1.000 | |
|  |  | 3rd day | LPS | | 1st day | | 1.3967 | 0.260 | 15.0 | | 5.374 | 0.001 | |
|  |  |  | LPS | | 2nd day | | 0.8333 | 0.260 | 15.0 | | 4.206 | 0.008 | |
|  |  |  | LPS | | 3rd day | | 0.8167 | 0.260 | 15.0 | | 4.820 | 0.011 | |
|  | LPS | 1st day | LPS | | 2nd day | | -0.5633 | 0.224 | 15.0 | | -2.518 | 0.355 | |
|  |  |  | LPS | | 3rd day | | -0.5800 | 0.224 | 15.0 | | -2.592 | 0.306 | |
|  |  | 2nd day | LPS | | 3rd day | | -0.0167 | 0.224 | 15.0 | | -0.074 | 1.000 | |

Note: df= degrees of freedom (using number of pairs of comparisons that were made).

**Table S4.** Bonferroni post-hoc test to detect significant differences in voluntary movement between *Xenopus laevis* treated with LPS (treatment group) and saline solution (control group) on the three test days for each variable analyzed.

| Post Hoc Comparisons - Treatment ✻ Test Days | | | | | | | | | | | |
| --- | --- | --- | --- | --- | --- | --- | --- | --- | --- | --- | --- |
|  | **Comparisons** | | | |  |  |  |  | | |  |
| Variables | **Treatment** | **Days** | **Treatment** | **Days** | **Mean Difference** | **SE** | **df** | | **t** | | ***p* bomferroni** |
|  |  |  |  |  |  |  |  | |  | |  |
| Total time in voluntary movement (min) | Control | 1st day | Control | 2nd day | 13.17 | 11.96 | 15.0 | | 1.101 | 1.000 | |
|  |  |  | Control | 3rd day | 15.33 | 11.96 | 15.0 | | 1.282 | 1.000 | |
|  |  |  | LPS | 1st day | 46.17 | 8.76 | 15.0 | | 5.268 | 0.001 | |
|  |  |  | LPS | 2nd day | 39.33 | 9.31 | 15.0 | | 4.224 | 0.011 | |
|  |  |  | LPS | 3rd day | 34.00 | 9.31 | 15.0 | | 3.651 | 0.035 | |
|  |  | 2nd day | Control | 3rd day | 2.17 | 11.96 | 15.0 | | 0.181 | 1.000 | |
|  |  |  | LPS | 1st day | 33.00 | 9.31 | 15.0 | | 3.544 | 0.044 | |
|  |  |  | LPS | 2nd day | 26.17 | 8.76 | 15.0 | | 3.986 | 0.038 | |
|  |  |  | LPS | 3rd day | 20.83 | 9.31 | 15.0 | | 4.237 | 0.013 | |
|  |  | 3rd day | LPS | 1st day | 30.83 | 9.31 | 15.0 | | 4.311 | 0.011 | |
|  |  |  | LPS | 2nd day | 24.00 | 9.31 | 15.0 | | 4.577 | 0.015 | |
|  |  |  | LPS | 3rd day | 18.67 | 8.76 | 15.0 | | 3.130 | 0.042 | |
|  | LPS | 1st day | LPS | 2nd day | -6.83 | 5.51 | 15.0 | | -1.241 | 1.000 | |
|  |  |  | LPS | 3rd day | -12.17 | 5.51 | 15.0 | | -2.210 | 0.646 | |
|  |  | 2nd day | LPS | 3rd day | -5.33 | 5.51 | 15.0 | | -0.969 | 1.000 | |
| Total time inside the shelter (min) | Control | 1st day | Control | 2nd day | 17.7 | 41.9 | 15.0 | | 0.422 | 1.000 | |
|  |  |  | Control | 3rd day | -11.2 | 41.9 | 15.0 | | -0.267 | 1.000 | |
|  |  |  | LPS | 1st day | -319.5 | 80.5 | 15.0 | | -3.970 | 0.018 | |
|  |  |  | LPS | 2nd day | -378.3 | 81.2 | 15.0 | | -4.657 | 0.005 | |
|  |  |  | LPS | 3rd day | -439.5 | 81.2 | 15.0 | | -5.409 | 0.001 | |
|  |  | 2nd day | Control | 3rd day | -28.8 | 41.9 | 15.0 | | -0.688 | 1.000 | |
|  |  |  | LPS | 1st day | -337.2 | 81.2 | 15.0 | | -4.150 | 0.013 | |
|  |  |  | LPS | 2nd day | -396.0 | 80.5 | 15.0 | | -4.920 | 0.003 | |
|  |  |  | LPS | 3rd day | -457.2 | 81.2 | 15.0 | | -5.627 | < .001 | |
|  |  | 3rd day | LPS | 1st day | -308.3 | 81.2 | 15.0 | | -3.795 | 0.026 | |
|  |  |  | LPS | 2nd day | -367.2 | 81.2 | 15.0 | | -4.519 | 0.006 | |
|  |  |  | LPS | 3rd day | -428.3 | 80.5 | 15.0 | | -5.322 | 0.001 | |
|  | LPS | 1st day | LPS | 2nd day | -58.8 | 107.0 | 15.0 | | -0.550 | 1.000 | |
|  |  |  | LPS | 3rd day | -120.0 | 107.0 | 15.0 | | -1.122 | 1.000 | |
|  |  | 2nd day | LPS | 3rd day | -61.2 | 107.0 | 15.0 | | -0.572 | 1.000 | |
| Number of breaths (count) | Control | 1st day | Control | 2nd day | 90.000 | 19.1 | 15.0 | | 4.7061 | 0.004 | |
|  |  |  | Control | 3rd day | 104.000 | 19.1 | 15.0 | | 5.4381 | 0.001 | |
|  |  |  | LPS | 1st day | 125.167 | 19.2 | 15.0 | | 6.5129 | < .001 | |
|  |  |  | LPS | 2nd day | 112.167 | 19.7 | 15.0 | | 5.6940 | < .001 | |
|  |  |  | LPS | 3rd day | 103.667 | 19.7 | 15.0 | | 5.2625 | 0.001 | |
|  |  | 2nd day | Control | 3rd day | 14.000 | 19.1 | 15.0 | | 0.7321 | 1.000 | |
|  |  |  | LPS | 1st day | 35.167 | 19.7 | 15.0 | | 1.7852 | 1.000 | |
|  |  |  | LPS | 2nd day | 22.167 | 19.2 | 15.0 | | 1.1534 | 1.000 | |
|  |  |  | LPS | 3rd day | 13.667 | 19.7 | 15.0 | | 0.6938 | 1.000 | |
|  |  | 3rd day | LPS | 1st day | 21.167 | 19.7 | 15.0 | | 1.0745 | 1.000 | |
|  |  |  | LPS | 2nd day | 8.167 | 19.7 | 15.0 | | 0.4146 | 1.000 | |
|  |  |  | LPS | 3rd day | -0.333 | 19.2 | 15.0 | | -0.0173 | 1.000 | |
|  | LPS | 1st day | LPS | 2nd day | -13.000 | 20.3 | 15.0 | | -0.6417 | 1.000 | |
|  |  |  | LPS | 3rd day | -21.500 | 20.3 | 15.0 | | -1.0613 | 1.000 | |
|  |  | 2nd day | LPS | 3rd day | -8.500 | 20.3 | 15.0 | | -0.4196 | 1.000 | |

Note: df= degrees of freedom (using number of pairs of comparisons that were made).
